# Matching Inhaler Devices with Patients: The Role of the Primary Care Physician

**DOI:** 10.1155/2018/9473051

**Published:** 2018-05-23

**Authors:** Alan Kaplan, David Price

**Affiliations:** ^1^Family Physician Airways Group of Canada, University of Toronto, Toronto, ON, Canada; ^2^University of Aberdeen, Aberdeen, UK; ^3^Observational & Pragmatic Research Institute, Singapore

## Abstract

Poor inhaler technique and nonadherence impair the efficacy of medications for asthma and chronic obstructive pulmonary disease (COPD). A range of factors, including age, dexterity, inspiratory capacity, cognitive ability, health literacy, and ethnicity, can impact a patient's ability and intention to use their device. Treatment success can also be influenced by patient preferences and perceptions. Therefore, it is important that healthcare professionals effectively match inhaler devices to individual patients' needs and abilities and empower patients by including them in treatment decisions. Physicians must, therefore, fully understand the characteristics of each device, as well as their patients' demographic characteristics and comorbidities. Following device selection, patient training and education, including a physical demonstration of the device, are key to eliminate any critical errors that may impact on health outcomes. Inhaler technique should be frequently rechecked. This review will examine the important role of primary care providers in the selection of appropriate inhaler devices and provision of training for patients with COPD and asthma to optimize correct inhaler use and adherence. An overview of the key features of available devices and of the factors to consider when selecting devices will be provided in the context of current asthma and COPD guidelines.

## 1. Introduction

Asthma and chronic obstructive pulmonary disease (COPD) are chronic inflammatory pulmonary diseases that affected 339 and 252 million people, respectively, worldwide in 2016 [1]. Both disorders cause airflow limitation, and inhaled medications—including inhaled corticosteroids, short- and long-acting *β*_2_ agonists, and antimuscarinic drugs—are central to their management [2, 3]. Inhaled therapy ensures rapid and direct delivery to the site of pathology and can be provided to patients via a range of devices, including nebulizers, pressurized metered-dose inhalers (pMDIs), dry powder inhalers (DPIs), and soft mist inhalers (SMIs). Despite the wide availability of these devices, poor inhaler technique and nonadherence to inhaled therapy has a marked effect on the therapeutic benefit of medication for asthma and COPD [4]. Studies consistently report that many patients with asthma and COPD do not use their inhaler devices correctly [5, 6]. A recent systematic literature review and meta-analysis found that incorrect technique is common across devices, with up to 100% of patients demonstrating at least one error [5]. Moreover, up to 92% of patients experience critical errors, that is, one that may impact the effectiveness of the delivered drug [5]. Indeed, medications for asthma and COPD cannot be effective if they do not reach the airways they are intended to target [7, 8]. Poor inhaler technique stems in part from the fact that the purpose and how to use inhalation devices are often poorly understood by patients, which can also lead to a reluctance to use the prescribed devices [9]. The variety of device types prescribed to patients can cause additional confusion and further impede effective use [10]. Due to these various reasons, poor adherence is common, with 50% or more of patients with asthma and COPD not taking their inhaled therapy as prescribed or instructed [11, 12]. Nonadherence can further perpetuate poor technique and can lead to costly exacerbations and worsening disease [11, 12].

It has been demonstrated that a shared-care approach (where patient goals and preferences are accommodated) and comprehensive patient education, including device training, can improve outcomes [13, 14]. However, even with training, not all patients are able to use their inhalers correctly [15]. For instance, some devices are unsuitable for use in certain patients (e.g., the patient may have an inability to hold the device correctly, or their instinctive technique may not match that of the prescribed device). Therefore, ensuring a match between patient and device at the outset of treatment is critical.

In this regard, as a common first point of contact and a prescriber of inhaled medications, the primary care physician has a vital role in optimizing outcomes for patients with asthma or COPD by selecting inhaler devices that are appropriate for individual patients. Furthermore, they are best placed to encourage the patient to participate in decisions about their treatment, as well as to help in providing patient education and training.

This review will examine the important role of primary care providers in the selection of appropriate inhaler devices for patients with COPD and asthma, specifically how matching the patient's inhaled therapy closely to his or her needs can improve inhaler technique and therefore their adherence to therapy and patient outcomes.

## 2. The Right Device for the Right Patient

In order to select the device that is most appropriate for a particular patient, it is important to recognize that a patient's ability to use a device may be influenced by a range of factors, including age, ethnicity, dexterity, and inspiratory capacity. Old or young age can be associated with a variety of elements to consider. This is pertinent as asthma is the most common chronic disease among children [16], and the prevalence of asthma and COPD increases with age [17, 18]. Older age, for example, increases the likelihood of comorbid conditions that may impact device selection [18, 19]. Physical issues including weakness, impaired dexterity, declining vision, poor hearing, and low inspiratory rates may impact on a patient's ability to use a device [20]. Declining cognitive function in the elderly can also impair the ability to master and recall techniques, as can cognitive and mood disorders, which are common comorbidities for asthma and COPD [18, 21]. Similarly, inhaler technique in children with asthma is generally very poor [22]. Despite being taught the correct technique, inhaler use is difficult for children and correct inhalation technique can deteriorate over time [23]. Furthermore, a child's ongoing adherence to inhaled treatment can also be influenced by a parent's participation in their disease management [24]. Looking more broadly, health literacy or language barriers may also influence device use by impacting a patient's ability to understand inhaler instructions [25]. If a patient fails to achieve symptomatic control due to poor inhaler technique, they may stop using their inhaler completely [20]. In addition to such unintentional nonadherence, patients may intentionally refuse to use a device. For example, commitment to inhaled medication can be affected by perceived social stigma surrounding device use, which has been shown to be an issue for adolescents [24]. Finally, the factors that contribute to adherence and impact upon successful treatment appear to vary by ethnic group, requiring further adaptation of approaches based on a patient's background [26, 27].

**Figure 1:**
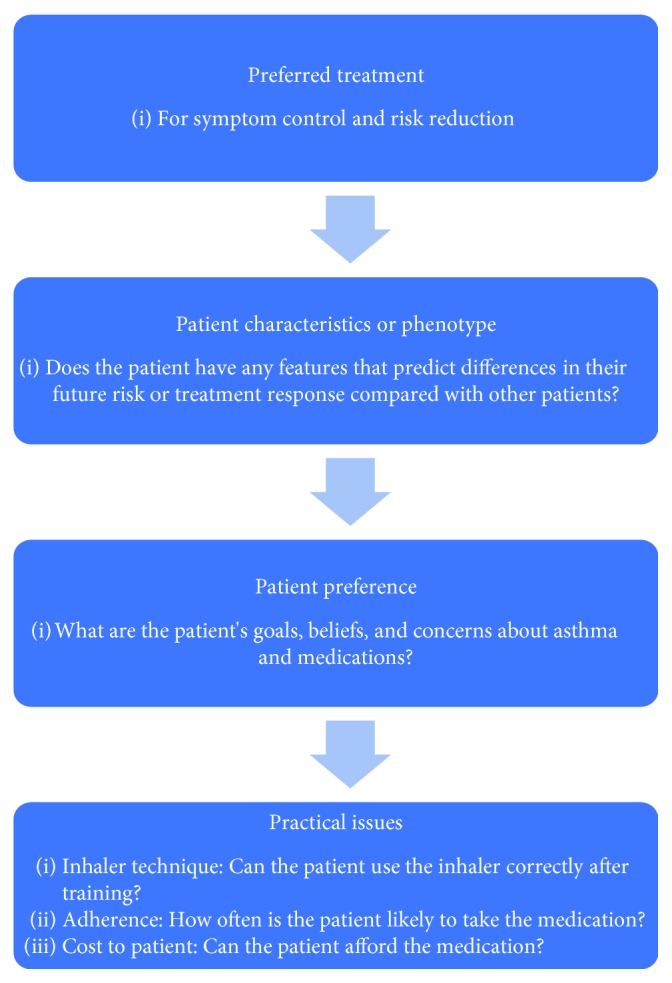
Overview of factors to consider when choosing controller options for individual patients [2].

To increase the likelihood of treatment success and adherence to therapy, it is crucial to match the device to the patient [25]. Current guidance recommends that “treatment decisions should [take] into account any patient characteristics or phenotype that predict the patient's likely response to treatment, together with the patient's preferences and practical issues” [2] (Figure 1). To do this, practitioners first need to understand the devices that are available to patients, their key characteristics, and how these might impact patients' preference and practice (Table 1).

### 2.1. Pressurized Metered-Dose Inhalers (pMDIs)

Recent innovations have enhanced the functionality of pMDIs, which were the first handheld inhalation device to be developed. However, common to all pMDIs are a pressurized canister of drug in solution or suspension, a chamber for producing an aerosol, and a mouthpiece for inhalation [28]. For use of first-generation, push-activated pMDIs, coordination of breathing and actuation is required. Poor coordination leads to reduced drug delivery, suboptimal disease control, and increased inhaler use. The recent CRITical Inhaler mistaKes and Asthma controL (CRITIKAL) study, which analyzed inhaler errors in 3660 patients who were using DPIs and pMDIs found that poor coordination (actuation before inhalation) was common in users of pMDIs (made by approximately a quarter of patients) and was associated with uncontrolled asthma [6]. Issues with poor coordination can be overcome by employing a spacer or a valved holding chamber (which allows inhalation over several intakes) [28]. However, these can be bulky, impacting portability [29]. Moreover, as an electrostatic charge can build up and decrease output in certain spacers, it has been recommended that priming doses of drug are used to deposit on the spacer walls or detergents can be used to coat the walls and reduce the charge [30, 31]. These issues can be mitigated with the use of nonelectrostatic spacers, such as the AeroChamber® Plus (Trudell Medical International) or Vortex® (PARI Respiratory Equipment) [32]. Newer models of MDIs are breath-actuated, reducing the dependency on coordinated inhalation and actuation [28], and are associated with better clinical asthma outcomes [33]. However, it is worth noting that the most frequent error consistently reported among pMDI users is failure to inhale slowly and deeply, although this may not significantly impact patient outcomes [6, 34].

### 2.2. Dry Powder Inhalers (DPIs)

DPIs deliver medication to the lungs in the form of a dry powder via the airstream created when the patient inhales through the device [28]. These devices are breath-actuated mitigating the problems with coordination that can arise with some pMDIs, and thus many patients find them easier to use [20]. However, many DPIs require a certain rate of inspiratory flow, making them unsuitable for patients with severe conditions and in certain emergency situations [20, 28]. The CRITIKAL study confirmed the importance of inspiratory effort, as insufficiently fast and forceful inhalation was associated with uncontrolled asthma occurring in roughly one-third of patients using DPIs [6]. To remove the reliance on inspiratory effort, device innovation has led to the development of “active,” power-assisted DPIs that use an energy source to disperse the drug; however, such devices are more costly [35]. There are a number of different types of DPI, with each requiring different techniques for use, and this can lead to confusion among users if devices are interchanged [20, 28]. The three main systems are as follows: capsule-based, where patients load a capsule containing the powered formulation into the device before each use; disposable devices containing a premetered single dose; or multiple-dose inhalers that either have a built-in mechanism to meter a single dose from a reservoir with each actuation or that deliver individual doses from premetered replaceable blisters [36]. For capsule devices, the patient must continue or repeat inhalation until the capsule is emptied, which can result in dose variability [36].

### 2.3. Soft Mist Inhalers (SMIs)

The SMI is the newest type of device, launched in 2007 when the Respimat® Soft Mist™ Inhaler (Boehringer Ingelheim) successfully gained approval for use in the European Union. The device uses spring power to provide treatment via a slow-moving fine liquid aerosol [20, 28]. It was designed to produce a higher fraction of fine and extra-fine particles (defined as 2.1–5 *μ*M and <2.1 *μ*M, resp. [37]), compared with most pMDIs and DPIs as well as to provide increased flexibility for synchronization between actuation and inhalation through the slow movement of the mist generated [38, 39]. Regional lung deposition varies according to the aerosol particle size, with particles of 2–6 *μ*M preferentially depositing in the central airways and those <2 *μ*M in the small airways and alveoli [37]. Therefore, the SMI results in higher lung (including smaller airways) and reduced oropharynx drug deposition compared with other device types [38, 39]. As the energy required to generate the aerosol is mechanical, and the softer longer lasting plume moves at a lower velocity compared with pMDIs, the inhalation effort to operate the SMI is lower.

### 2.4. Nebulizers

Nebulizers can be used in patients for whom handheld devices are unsuitable, including the very young, the elderly, and the acutely ill [20, 40]. They require comfortable tidal breathing and little coordination from the patient. However, they can be noisy, and some require an outside energy source [40]. Generally, they lack portability, require regular maintenance, are expensive, and result in longer treatment times [20]. However, new nebulizers have recently been developed to overcome some of these disadvantages, for example, the handheld Aeroneb® Go (Philips Healthcare), which is portable, compact, and silent [41].

### 2.5. Considerations for Device Selection

**Figure 2:**
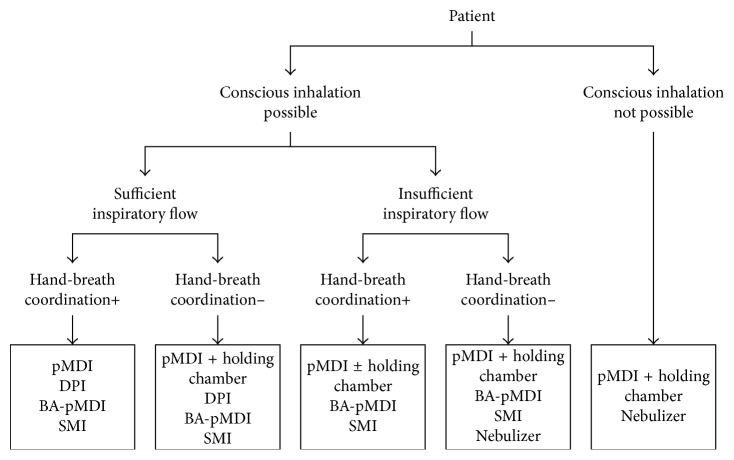
Device selection algorithm. Reproduced with permission from [28]. DPI, dry powder inhaler; pMDI, pressurized metered-dose inhaler; BA, breath-actuated; SMI, soft mist inhaler.

The different inhalation techniques required for the different available devices and inspiratory abilities, as well as patient dexterity and cognition, form the key considerations when selecting a device (Figure 2). To aid selection, the use of training devices/inspiratory flow meters (such as AIM™ (Aerosol Inhalation Monitor), In-Check DIAL, and 2-Tone trainer) can be used to assess the patient's inhalation technique and inspiratory ability, either when first prescribing a new device to a patient or during regular training and monitoring of inhaler use [34, 42]. However, an inspiratory flow assessment may not always be reflective of a patient's inspiratory ability in a real-world setting, and observation of instinctive inhaler technique can also indicate the type of inhaler that will be best suited to the patient. Moreover, some prescribed inhalation devices can indicate whether a patient is using them appropriately and has the required inspiratory capacity (e.g., Duaklir Genuair® (AstraZeneca UK Ltd.), where a control window changes to red from green when the patient has inhaled correctly), aiding both device choice and patient technique.

Current guidelines recommend that device selection should be made in consultation with the patient, who must be trained in inhaler device technique [2, 3]. Asthma and COPD treatment guidelines also state that inhaler technique must be regularly assessed during follow-up consultations [2, 3]. Moreover, the prescription of mixed inhalation device types should be avoided to prevent confusion [2]. A large retrospective observational study of patients with asthma in primary care demonstrated that over one year after a first inhaled corticosteroid prescription, patients prescribed the same device type for both controller and reliever therapy were significantly more likely to achieve asthma control and recorded significantly lower exacerbation rates than those prescribed mixed devices [43]. Indeed, several studies have shown that simultaneous use of different inhaler types is predictive of increased inhalation errors [10]. In addition, asking patients to switch devices can impact adherence to therapy. In a retrospective observational study of patients with COPD, multiple-inhaler users demonstrated lower adherence rates than single-inhaler users and were significantly more likely to discontinue therapy [44].

## 3. Patient Perceptions and Shared Decisions

Patients have their own perceptions or preferences regarding inhaler choice, and these can influence treatment success [28]. Device features such as simplicity, convenience, and overall experience are important to patients [28]. Patients have reported spontaneously ceasing inhaled therapy due to perceived complexity and are likely to value device features associated with convenience such as ease of cleaning, comfort, and portability [28]. Beliefs surrounding treatment—for example, whether it will be ineffective, cause side effects, or should only be taken when “really needed”—may also affect a patient's willingness to adhere to therapy, as might issues with device use, such as taste and effect on the throat [28]. By including the patient in treatment decisions, it is possible to tailor device selection around such perceptions and preferences, as well as gaining an understanding of the patient's approach to treatment. Even if a different device seems to be a better match for the patient, it may be that they are reluctant to try a new device because they have become used to their existing inhaler. They may also be apprehensive when switching devices due to the requirement to acquire new skills [28]. Others will be encouraged by changing device if they are having a poor experience with current treatment. Indeed, lack of perceived benefit has been reported as leading patients to intentionally discontinue their therapy [45]. In either case, shared decision-making can empower the patient, and it has been shown to improve both adherence and health outcomes [13]. In a randomized controlled trial where clinicians and patients negotiated a treatment plan that accommodated patient goals and preferences, shared decision-making was associated with a higher cumulative dose of medication over a one-year period, a higher likelihood of well-controlled asthma, and better lung function [13]. It is therefore important that patients are encouraged to participate in treatment decisions and to express their expectations and concerns [2, 3].

## 4. Common Errors and Patient Education

Patient errors with inhaler technique are common and have been linked with poor outcomes in asthma and COPD [6, 9, 12]. The CRITIKAL study highlighted that errors relating to inspiratory effort were frequent in patients using DPIs and MDIs [6]. Inhalation was insufficiently fast and forceful in up to 38% of individuals using DPIs and was not slow or deep enough in 47% of patients using MDIs [6]. Errors common with both device types included not having the head tilted with chin up during inhalation and not breathing out to empty lungs before inhalation [6]. Many inhaler errors were associated with asthma symptom control; for example, insufficient inspiratory effort occurred frequently and was significantly associated with an increased likelihood of uncontrolled asthma in patients using DPIs [6]. For patients using MDIs, errors relating to device knowledge and second dose preparation were associated with uncontrolled asthma, as was actuation before inhalation [6]. A systematic review of inhaler use, covering 59,584 observed tests of technique, found that the overall prevalence of correct technique was 31% and that inhaler technique has not improved in 40 years [46].

Such studies emphasize the need for new approaches to patient education and training. A recent systematic review of educational inhalation technique interventions found that over 90% of studies included reported a significant improvement in inhaler technique after an educational intervention (although average follow-up time was short) [47]. Interventions in outpatient clinics performed best, indicating the importance of primary care providers [47]. The “teach-back” technique—where patients demonstrate their inhaler use after instruction—has been shown to be particularly beneficial [48]. As well as providing an effective initial educational intervention, continued support is vital [49, 50]. Indeed, the Global Initiative for Asthma (GINA) strategy advises that clinicians recheck inhaler technique frequently, as errors often recur within 4–6 weeks after initial training [2].

The primary care physician is best placed to address patients' perceptions and attitudes towards therapy, to individualize treatment choice, and to provide tailored education and device training to maximize adherence to treatment. It is also important that pharmacists are familiar with the device technique, as they are usually the last (and sometimes the most frequent) healthcare professional to be seen by patients before a device is used. Studies have demonstrated that they can provide effective skills training positively impacting disease control [50, 51]. As such, they play an important role in reinforcing inhaler technique and primary care physicians can recommend that their patients also ask their pharmacists any questions about device handling, especially when supplied with their own inhaler and a check-correct-confirm cycle can be conducted [50, 52]. While patient education is often effective, it is critical to remember that some patient groups will be unable to use certain devices despite receiving adequate training [21, 53], thereby reinforcing the importance of first matching the patient with the optimum device. Finally, it is essential that the selected device reaches the patient. While it is not commonplace in many countries, it should be stated that pharmacists should not amend device prescriptions and the switching of inhaler devices without an accompanying practitioner consultation should be avoided.

## 5. Case Study

- A 68-year-old woman who had been prescribed a tiotropium bromide, HandiHaler®, presents to her doctor with dyspnea. She was adherent to her medication, and cardiac etiologies were ruled out. Her spirometry showed GOLD stage 3 disease with a forced expiratory volume in 1 second (FEV_1_) of 48%.
- It was decided to step up her medication to a long-acting bronchodilator combination of *β*_2_-agonist (LABA)/muscarinic antagonist (LAMA), in the form of formoterol/aclidinium (Duaklir Genuair) administered twice a day. She was adherent, but her condition deteriorated. She felt depressed as her health worsened despite her medication change.
- We reviewed her inhaler technique. She was using the device upside down, which was corrected. She was also reminded of the twice-daily dosing, which she occasionally forgot.
- A repeat visit showed that the color window did not turn from green to red after actuation, indicating that she had insufficient inspiratory flow to activate it. She was converted to tiotropium/olodaterol, a Respimat device administered once daily. She had some difficulty loading the canister due to hand osteoarthritis, and the pharmacist agreed to load her medication for her on dispensing.
- She took two inhalations once daily each morning and was amazed at how much better she felt. Her exercise tolerance increased, and her mood improved.

## 6. Future Considerations

Primary care physicians have a key role to play in maximizing inhaled therapy adherence in asthma and COPD by ensuring that the patient is using a suitable inhaler device, and can learn and maintain inhaler technique. This requires an holistic approach to treatment optimization. Physicians are required to have a full understanding of the characteristics of each device, in addition to knowledge of their patient's physical and cognitive abilities. Moreover, physicians should understand cultural beliefs and perceptions around treatment, which may impact on adherence. The GINA guidelines outline strategies to ensure effective use of inhaler devices, which should serve as a basis for all primary care physicians prescribing these (Table 2). Essentially, they advocate four Cs, summarized below:

- Choose: choose the most appropriate inhaler device for the patient before prescribing
- Check: check inhaler device technique at every opportunity, including asking the patient to demonstrate their inhaler
- Correct: show the patient how to use the device correctly via a physical demonstration and recheck technique frequently
- Confirm: clinicians should be able to demonstrate correct inhaler technique. Skills training can be reinforced by pharmacists and nurses.

While algorithms are available to aid device choice [54], these tend to focus on inspiratory ability and basic physical traits. Practitioners should be aware that their use will need to be supplemented with an in-depth understanding of adherence and technique behaviors in key patient subgroups. Furthermore, it is evident that many healthcare professionals are themselves unable to demonstrate correct inhaler technique [55]. Given that the most effective patient training technique in correct inhaler use relies upon physical demonstration [49], priority must be given to providing effective training for healthcare professionals to enable them to effectively educate their patients. Integral to training for healthcare professionals should be an awareness of common mistakes and reasons for nonadherence, which can serve as a checklist in the provision of patient education [14]. It is also important to note that inhaler design is an advancing field. Devices are undoubtedly going to become “smarter,” providing feedback to the patient on adherence and technique. However, as outlined in a recent editorial commenting on a study by Sulaiman et al. [56], the authors highlighted that while biofeedback devices may improve adherence and, therefore, disease control, there is a relatively small impact [57]. Education and structured health plans remain hugely important, as will keeping up-to-date with training as these new devices become available [35].

## 7. Conclusions

Inhaled therapies are the cornerstone of asthma and COPD management. It is therefore vital that inhaler devices are prescribed that patients can and will use. Treatment decisions should be made in partnership with patients, and should take into consideration demographic characteristics and comorbidities to allow device choice to be individualized. As asthma and COPD control is suboptimal, due to patient errors with inhaler technique, common errors may serve as a checklist for physicians to aid inhaler technique training. Ultimately, healthcare providers who work with patients who use inhaled medications have an essential role in minimizing common patient errors and are therefore required to “choose, check, correct, and confirm” to ensure effective use of inhaler devices among their patients.

## Figures and Tables

**Table 1:** Advantages and disadvantages of the main types of inhalers.

Inhaler	Advantages	Disadvantages
pMDI	(i) Portable and compact	(i) Require coordination
	(ii) Multidose device	(ii) High deposition in mouth and oropharynx
	(iii) Metered dose	(iii) “Cold Freon” effect
	(iv) Established/familiar	(iv) Contain propellants
	(v) Available for most inhaled medications

pMDI + spacer	(i) Lower dependency on inspiratory effort	(i) Less portable than pMDI
	(ii) Easier to coordinate	(ii) Certain spacers may acquire electrostatic charge
	(iii) Higher lung deposition than pMDI	(iii) Additional cost to pMDI
	(iv) Reduced mouth and oropharynx deposition	(iv) Requires regular maintenance

BA-MDI	(i) Portable and compact	(i) Contain propellants
	(ii) Multidose device	(ii) “Cold Freon” effect
	(iii) Breath-actuated	(iii) Requires a moderate inspiratory effort

DPI	(i) Portable and compact	(i) Requires a minimum inspiratory effort
	(ii) Breath-actuated	(ii) May not be appropriate for emergency situations
	(iii) Does not contain propellants	(iii) Multiple designs (may be confusing for the patient)
	(iv) Multidose devices available	(iv) May be complicated to load

SMI	(i) Portable and compact	(i) Not breath-actuated
	(ii) Multidose device	(ii) Only one device currently available
	(iii) Lower dependency on inspiratory effort
	(iv) High fine-particle fraction
	(v) High lung deposition; low mouth and oropharynx deposition
	(vi) Does not contain propellants

Nebulizers	(i) Can be used at any age	(i) Most lack portability
	(ii) Can be used by acutely ill	(ii) Some require an outside energy source
	(iii) No specific inhalation technique required	(iii) Noisy
	(iv) Can be used to dispense drugs not available as pMDI or DPI	(iv) Can result in longer treatment times
		(v) Can be expensive

Adapted from [28]. BA-MDI, breath-actuated metered-dose inhaler; DPI, dry powder inhaler; pMDI, pressurized metered-dose inhaler; SMI, soft mist inhaler.

**Table 2:** Strategies to ensure effective use of inhaler devices [2].

Choose
(i) Choose the most appropriate inhaler device for the patient before prescribing. Consider the medication options, the available devices, patient skills, and cost
(ii) If different options are available, encourage the patient to participate in the choice
(iii) For pMDIs, use of a spacer improves delivery and (with ICS) reduces the potential for side effects
(iv) Ensure that there are no physical barriers, for example, arthritis, that limit the use of the inhaler
(v) Avoid use of multiple different inhaler types where possible, to avoid confusion

Check
(vi) Check inhaler technique at every opportunity
(vii) Ask the patient to show you how they use their inhaler (do not just ask if they know how to use it)
(viii) Identify any errors using a device-specific checklist

Correct
(ix) Show the patient how to use the device correctly with a physical demonstration, for example, using a placebo inhaler
(x) Check technique again, paying attention to problematic steps. You may need to repeat this process 2-3 times
(xi) Only consider an alternative device if the patient cannot use the inhaler correctly after several repeats of training
(xii) Recheck inhaler technique frequently. After initial training, errors often recur within 4–6 weeks

Confirm
(xiii) Clinicians should be able to demonstrate correct technique for each of the inhalers they prescribe
(xiv) Pharmacists and nurses can provide highly effective inhaler skills training

Reproduced with permission from [2]. pMDI, pressurized metered-dose inhaler; ICS, inhaled corticosteroids.
